# Study protocol – elucidating the neural correlates of functional remediation for older adults with bipolar disorder

**DOI:** 10.3389/fpsyt.2023.1302255

**Published:** 2024-01-17

**Authors:** Laura Montejo, Brisa Sole, Lydia Fortea, Esther Jimenez, Anabel Martinez-Aran, Eloy Martinez-Heras, Jose Sanchez-Moreno, Maria Ortuño, Jose Pariente, Aleix Solanes, Carla Torrent, Enric Vilajosana, Michele De Prisco, Eduard Vieta, Joaquim Radua

**Affiliations:** ^1^Bipolar and Depressive Disorders Unit, Hospital Clinic de Barcelona, Barcelona, Spain; ^2^Institut d’Investigacions Biomèdiques August Pi i Sunyer (IDIBAPS), Barcelona, Spain; ^3^Institute of Neurosciences (UBNeuro), Barcelona, Spain; ^4^Centro de Investigación Biomédica en Red de Salud Mental (CIBERSAM), Instituto de Salud Carlos III, Madrid, Spain; ^5^Departament de Medicina, Facultat de Medicina i Ciències de la Salut, Universitat de Barcelona, Barcelona, Spain; ^6^Departament de Psicologia Clínica i Psicobiologia, Facultat de Medicina i Ciències de la Salut, Universitat de Barcelona, Barcelona, Spain; ^7^Neuroimmunology and Multiple Sclerosis Unit and Laboratory of Advanced Imaging in Neuroimmunological Diseases (ImaginEM), Hospital Clinic Barcelona, Fundació de Recerca Clínic Barcelona-IDIBAPS, Barcelona, Spain; ^8^Magnetic Resonance Image Core Facility, Institut d'Investigacions Biomediques August Pi i Sunyer (IDIBAPS), Barcelona, Spain

**Keywords:** older adults, bipolar disorder, neuroimaging, functional remediation, functional outcome

## Abstract

**Introduction:**

Beyond mood abnormalities, bipolar disorder (BD) includes cognitive impairments that worsen psychosocial functioning and quality of life. These deficits are especially severe in older adults with BD (OABD), a condition expected to represent most individuals with BD in the upcoming years. Restoring the psychosocial functioning of this population will thus soon represent a public health priority. To help tackle the problem, the Bipolar and Depressive Disorders Unit at the Hospital Clínic of Barcelona has recently adapted its Functional Remediation (FR) program to that population, calling it FROA-BD. However, while scarce previous studies localize the neural mechanisms of cognitive remediation interventions in the dorsal prefrontal cortex, the specific mechanisms are seldom unknown. In the present project, we will investigate the neural correlates of FR-OABD to understand its mechanisms better and inform for potential optimization. The aim is to investigate the brain features and changes associated with FROA-BD efficacy.

**Methods:**

Thirty-two individuals with OABD in full or partial remission will undergo a magnetic resonance imaging (MRI) session before receiving FR-OABD. After completing the FR-OABD intervention, they will undergo another MRI session. The MRI sessions will include structural, diffusion-weighted imaging (DWI), functional MRI (fMRI) with working memory (n-back) and verbal learning tasks, and frontal spectroscopy. We will correlate the pre-post change in dorsolateral and dorsomedial prefrontal cortices activation during the n-back task with the change in psychosocial functioning [measured with the Functioning Assessment Short Test (FAST)]. We will also conduct exploratory whole-brain correlation analyses between baseline or pre-post changes in MRI data and other clinical and cognitive outcomes to provide more insights into the mechanisms and explore potential brain markers that may predict a better treatment response. We will also conduct separate analyses by sex.

**Discussion:**

The results of this study may provide insights into how FROA-BD and other cognitive remediations modulate brain function and thus could optimize these interventions.

## 1 Introduction

Bipolar disorder (BD) is a chronic mental illness characterized by the presence of manic/hypomanic and depressive episodes alternated with partial or total euthymic periods. This disease frequently presents cognitive deficits mainly affecting attention, executive function, memory, and processing speed (1–3). Unfortunately, these cognitive deficits result in functional impairment and an overall decrease in quality of life, issues that are usually not adequately addressed by traditional pharmacological treatments (4).

In recent years, cognitive impairment has emerged as a key treatment priority in BD. Cognitive Remediation (CR) has shown consistent cognitive benefits in BD (5) with improvements in several cognitive domains, especially attention, verbal memory, and executive functions (6, 7). In fact, it has also been found that cognitive improvements contribute to functional improvement, demonstrating a translation of the therapeutic benefits into the patients’ daily life (8). However, most CR interventions for BD are designed for younger people, overlooking the specific and relevant characteristics of Older Adults with Bipolar Disorder (OABD). According to the latest definition of the International Society for Bipolar Disorders (ISBD) OABD Task Force, OABD is defined as people with BD older than 50 years of age (9). In recent years, special emphasis has been placed on considering OABD as a particular subgroup of the population, since it has been reported that they exhibit specific characteristics, such as a different clinical profile, lower functioning, and the presence of higher rates of somatic comorbidities compared to younger patients (10–12). These findings reinforce the idea of developing and applying specific clinical and treatment strategies adapted for this group of patients.

OABD is considered a vulnerable population to cognitive impairment (13). A recent metanalysis has demonstrated that OABD exhibit significantly worse performance in almost all cognitive domains, especially in the memory domain, but also in attention, working memory, executive functions, and processing speed, compared to healthy controls (14). Impairments in different cognitive domains have also been associated with poorer psychosocial functioning in OABD (15–17). Regardless, the relevance of functional decline, cognitive impairment and reduced quality of life emphasizes the importance of developing and optimizing therapeutic strategies aimed at remediating cognition and functioning in OABD.

However, most interventions that have demonstrated effectiveness in improving cognitive performance have the inconvenience that, on the one hand, are designed for older adults with different psychiatric diagnoses or, on the other hand, are tailored for BD without addressing this age range specifically. This limitation restricts the effectiveness and treatment response in these patients. To maximize therapeutic benefits for this age group, it is crucial to develop interventions tailored to their specific needs (18). Given the scarce evidence of treatments aimed at improving or enhancing cognitive function in OABD, the original “Functional Remediation” (FR) cognitive program for BD (19) was recently adapted to the specific characteristics of OABD, thus creating the FROA-BD program (20). The efficacy of the original FR program was tested in a randomized controlled trial (RCT) that showed that it improved psychosocial functioning (19) and its effects were maintained over time (21). FR also showed to improve verbal memory in those participants showing significant cognitive impairment (22) and was also effective for patients with subsyndromal symptoms (23) as well as for patients with bipolar II type (24). Therefore, the FROA-BD intervention introduces the novelty of attempting to fill this gap in treatment options for this patient group. The FROA-BD focuses on improving cognitive performance, functionality, and quality of life, but also addresses emotion management, social skills, autonomy, strategies for daily living, healthy lifestyle habits, and training in digital competencies.

Understanding the neural correlates of cognitive impairment and cognitive remediation is crucial, as it provides insights into the brain mechanisms underlying cognitive processes and facilitates targeted interventions to enhance cognitive function. In studies investigating the neuronal basis of cognitive impairment in BD, regarding structural brain abnormalities, a greater left dorsomedial prefrontal thickness and a lower cerebral white matter (WM) volume have been associated with cognitive impairment (25). In addition, the most consistent findings regarding neural correlates for cognitive impairment also suggest a failure to activate task-relevant prefrontal regions and to deactivate the default mode network (25, 26). The ISBD Targeting Cognition Task Force recommended using neuroimaging in intervention trials to optimize cognitive remediation (27). According to these recommendations, we need to include task-related functional Magnetic Resonance Imaging (fMRI) assessments given that they allow us to identify changes in cognition-relevant function and structure. Moreover, given the paucity of structural neuroimaging intervention studies further studies are needed to clarify the value of these techniques as biomarkers for treatments targeting cognition. In recent years, some interesting studies have investigated brain effects of similar cognitive remediation (CR) programs using Magnetic Resonance Imaging (MRI) and whether baseline MRI can predict improvement in cognition after receiving CR. However, results are still limited, and the studies only have investigated younger patients. One study, which combined participants with BD and depression, found a correlation between improvement in working memory and verbal memory and activation in prefrontal, parietal, and hippocampal regions during n-back working memory and verbal memory tasks (28). A more recent study focused on BD found that improvement in executive functions correlated with dorsolateral prefrontal cortex activation during the n-back working memory task (29). Furthermore, both baseline hypoactivation and reduced dorsolateral prefrontal cortical thickness predicted improved executive functions after 10 weeks of CR intervention (29–31). In this sense, it has been suggested that the association between task difficulty and dorsolateral prefrontal response seems to be based on a bell-shaped load-response curve (25). According to this integrative model, patients show dorsolateral prefrontal hyperactivation when task performance remains at an average difficulty level. In contrast, dorsolateral prefrontal hypoactivation is observed when difficulty decreases compared to controls. As mentioned before, OABD is a population who has received less research, so no consistent findings have been detected in studies investigating neural substrates implicated in cognitive impairment (32) and no studies have examined the neural changes in response to cognitive remediation.

Consequently, there is a lack of a validated theoretical framework for the underlying neurobiological mechanisms of cognitive improvement and cognitive remediation in OABD. The neural mechanisms of cognitive remediation therapies constitute novel strategies to optimize these interventions (32). Neuroimaging will likely reveal neurocircuit-based biomarkers that may inform key general or individual steps to increase the efficacy or personalization of FROA-BD.

Thus, the overarching aim of this study is to investigate the functional brain correlates of this intervention.

### 1.1 Hypotheses

The main hypothesis of this study is that, after completing the FROA-BD program, patients will show a correlation between the changes in their dorsolateral or dorsomedial prefrontal cortex activation during a working memory task (compared to baseline) and improved psychosocial functioning.

The secondary, explorative hypotheses are that there exist correlations between changes in other MRI-derived brain signals and cognition and psychosocial improvements and between baseline MRI-derived brain signals and cognition and psychosocial improvements. Based on the above studies finding neural correlates of cognition remediation in BD, we hypothesize that the correlations will involve structural MRI and task-based fMRI. However, due to the study’s novelty, we do not have clear hypotheses about their specific brain locations. That said, previous research on patients with psychotic and bipolar disorders indicates increased cortical thickness in the frontal and parietal cortices at baseline, correlating with enhanced cognitive improvements following CR (30, 31, 33, 34). Thus, we may tentatively hypothesize that increased cortical thickness in these regions will predict significant improvements in cognitive function after FROA-BD.

Finally, the study also hypothesizes that there may be potential differences depending on sex.

### 1.2 Objectives

The main objective is to correlate the pre-post changes in the activation of the dorsolateral and dorsomedial prefrontal cortices during the working memory n-back task (2-back vs. baseline) and pre-post changes in psychosocial functioning assessed with the Functioning Assessment Short Test (FAST).

Secondary, explorative objectives are to correlate (a) pre-post changes in MRI data with pre-post changes in cognition and psychosocial functioning outcomes; and (b) baseline MRI data with pre-post changes in cognition and psychosocial functioning outcomes. For the secondary objectives, we plan to conduct comprehensive whole-brain correlation analyses (corrected for multiple testing) and use several MRI modalities (regional tissue volume, cortical thickness, and surface area; fractional anisotropy and diffusivity measures, activation during the working memory n-back task and a verbal memory task; and frontal spectroscopy).

Finally, we will repeat all the analyses on those results that reach statistical significance according to sex to explore potential differences depending on sex.

## 2 Methods and analysis

### 2.1 Study design, participants, and procedure

This study is an addition to the NCT05186337 clinical trial. We will conduct a prospective longitudinal observational MRI study. We will invite older adult patients diagnosed with BD type I or II who are about to receive FROA-BD at the Bipolar and Depressive Disorder Unit at the Hospital Clinic of Barcelona to participate. The sample will comprise at least 32 patients (50% males and 50% females). Inclusion criteria will include: (a) aged 60 or over; (b) diagnosis of BD type I or type II according to the Diagnostic and Statistical Manual of Mental Disorders (DSM-5) (35); (c) full or partial remission at the time of assessment, defined as scores ≤14 on the Hamilton Assessment of Depression Scale (HDRS) (36, 37) and ≤ 10 on the Young Mania Rating Scale (YMRS) (38, 39); (d) presence of functional impairment defined as a total score ≥ 11 on the FAST (40); and (e) written informed consent to participate in the study. Exclusion criteria will include: (a) estimated intelligence quotient (IQ) lower than 85; (b) having received a structured psychological intervention within the last 6 months; (c) presenting any central nervous system condition, other than mental disorders, that may affect neuropsychological performance or any physical condition that may hamper participation or assimilation of the contents of the intervention (e.g., severe visual or hearing impairment); (d) presence of any current comorbid psychiatric condition; (e) history of cranioencephalic trauma; and (f) having received electroconvulsive therapy 6 months before the inclusion study.

After consenting, participants will undergo a demographic, clinical, functional, and neuropsychological assessment and an MRI session. Afterward, patients will receive the 32 sessions of FROA-BD throughout 4 months while continuing their usual pharmacological treatment. Finally, after finishing the FROA-BD, patients will undergo a second clinical, functional, and neuropsychological assessment and a second MRI session. For more details on the FROA-BD protocol and program description, see Montejo et al. (20).

Discontinuation criteria will include (a) withdrawal of consent by the participant; (b) significant clinical relapse or hospitalization for any episode; and (c) not attending more than eight sessions during the intervention.

### 2.2 Sample size estimation

We aim to include 28 participants because previous studies with 23–28 participants found effects (29–31). In addition, with this sample size, we would have ~80% power to detect moderate correlations (r 0.56) after correcting for multiple testing (correlations in three ROIs: left dorsolateral, bilateral dorsomedial, and right dorsolateral prefrontal cortices). Note that this sample size does not include those patients who, even if consenting to participate, leave the study before finishing the baseline assessment and MRI acquisition.

As we anticipate the possibility of participant attrition during the follow-up, we plan to enroll 32 participants (i.e., ~15% more) to counteract the potential dropouts.

### 2.3 Clinical and neuropsychological assessment

We will use a semistructured clinical interview based on the SCID-5 (41) complemented with a revision of medical records to assess baseline demographic and clinical variables. To assess clinical symptomatology, functional outcome, cognitive reserve, quality of life and well-being, and medical comorbidity, we will use the HDRS, the YMRS, and other standard scales shown in Table 1. A trained neuropsychologist will then administer a comprehensive neuropsychological battery of tests to evaluate the following cognitive domains: attention, processing speed, working memory, verbal learning and memory, visual memory, executive functions, language, and visuospatial skills (Table 1). The selected instruments are considered reliable tools to assess cognition in BD, and they are included in the consensus proposed by the International Society for Bipolar Disorders (ISBD) (42). Moreover, the cognitive assessment will also include two cognitive screening instruments [the Minimental Status Examination (MMSE) (43) and the Spanish version of the Screen for Cognitive Impairment in Psychiatry (SCIP-S) (44, 45)], and a scale for gathering subjective cognitive complaints [the Cognitive Complaints in Bipolar Disorder Rating Assessment (COBRA) (46)].

**Table 1:** Clinical and cognitive measurements at baseline and post-treatment.

		Baseline	Post-treatment
Clinical symptomatology	HDRS		
	YMRS		
Medical comorbidity	CIRS-G		
Psychosocial Functioning	FAST		
Well-being and quality of life	SF-36		
	WHO-5		
Cognitive reserve	CRASH		
Cognition	MMSE		
	SCIP-S		
	Vocabulary (WAIS-III)		
	Arithmetic (WAIS-III)		
	Symbol Search (WAIS-III)		
	Digit-Symbol coding (WAIS-III)		
	Digits (WAIS-III)		
	Letter-Number Sequencing (WAIS-III)		
	WSCT		
	SCWT		
	ROCF		
	CPT-II		
	TMT-A		
	TMT-B		
	CVLT		
	F-A-S (COWAT)		
	Animal Naming (COWAT)		
	Boston Naming Test		
	Juice Line Orientation-form H		
	COBRA		

CIRS-G, Cumulative Illness Rating Scale-Geriatrics; COBRA, Cognitive Complaints in Bipolar Disorder Rating Assessment; COWAT, Controlled Oral Word Association Test; CPT, Continuous Performance Test; CRASH, Cognitive Reserve Assessment Scale in Health; CVLT, California Verbal Learning test; FAST, Functional Assessment Short Test; fMRI, Functional Magnetic Resonance Imaging; HDRS, Hamilton Depression Rating Scale; MRI, Magnetic Resonance Imaging; MMSE, Mini-Mental State Examination; ROCF, Rey Osterrieth Copy figure; SCIP, Screen for Cognitive Impairment in Psychiatry; SCWT, Stroop Color Word Test; TMT-A, Trail Making Test part A; TMT-B, Trail Making Test part B; YMRS, Young Mania Rating Scale; WAIS-III, Wechsler Adults Intelligence Scale 3rd edition; WCST, Wisconsin Card Sorting Test; WHO-5, World Health Organization-Five Well-Being Index. Color shading means when the clinical and cognitive measurements at baseline and post-treatment are administered.

The post-intervention assessment will be briefer (Table 1), comprising the assessment of clinical and functional outcomes, quality of life, and subjective cognitive complaints, but a substantially brief neuropsychological assessment. The latter will only include the alternative form of the cognitive screening tool (SCIP) (45) and a few tests to assess attention and executive functions to avoid potential learning effects, given the short period between baseline and post-intervention assessment. For more details about all the assessment tests, see Table 1.

### 2.4 Intervention description

The FROA-BD program consists of a group intervention (approximately eight participants per group) with 90-min sessions twice weekly for 16 weeks. It aims to improve psychosocial functioning by targeting neurocognitive functions such as attention, memory, executive functions, language, and visuospatial skills. It also addresses healthy lifestyle habits, emotional management, social skills, and training in digital competencies. It includes 30 sessions for patients and two sessions for relatives or caregivers. The sessions provide different kinds of strategies to improve psychosocial functioning, focusing on cognitive training of the different cognitive functions. It is important to highlight that, in the FROA-BD intervention, patients aged 60 or older will be included instead of 50 years old as the ISBD defined OABD population; this is because the original FR program established a cut-off point below 60, therefore, we tried to cover this usually excluded age range from traditional RCTs.

### 2.5 MRI acquisition and functional tasks

MRI acquisitions will be conducted on a Siemens Magnetom Prisma Fit 3 T device at Hospital Clínic of Barcelona and will last approximately 45 min. We will instruct participants to remain still and place pads around their heads to minimize head movement.

The MRI acquisition protocol will include a high-resolution T1-weighted structural Three-dimensional Magnetization-Prepared Rapid Acquisition with Gradient Echo (3D-MPRAGE) sequence (repetition time (TR) 2,300 ms; echo time (TE) 3 ms; inversion time (TI) 900 ms; flip angle 9°; 240 sagittal slices with 1 mm×0.94 mm×0.94 mm voxel size and 256 × 256 matrix size), a multi-shell DWI sequence (90 diffusion encoding directions and two different phase encoding directions (AP and PA); b-values 1,000, 2000, and 3,000 s/mm^2^; five b0 volumes; TR 5400 ms; TE 113 ms; 100 transverse slices with 1.5 mm isotropic voxel size and 150 × 150 matrix size), a single voxel spectroscopy (SVS) localized in the left dorsomedial prefrontal cortex (with and without water suppression, with 96 and 16 acquired averages, respectively; TR 3000 ms; TE 30 ms; 20x30x15mm voxel size), two gradient-echo echo-planar (EPI) sequences for the cognitive tasks (TR 1500 ms; TE 37 ms; 72 transverse slices with 2 mm isotropic voxel size and 104 × 104 matrix size), and their corresponding gradient field mapping (GFM) to estimate and correct susceptibility artifacts caused by lack of homogeneity in the field. We will inspect the images visually to detect anomalies or artifacts.

#### 2.5.1 N-Back paradigm

The sequential-letter version of the n-back working memory task (duration: 8 min 42 s) evaluates two levels of working memory load (1-back and 2-back) in a block-design manner. During the blocks, a projector displays a single letter on the screen every 2 s (1 s on, 1 s off). Each block consists of 24 letters, including five randomly located targets. In the 1-back blocks, the letters are color-coded in green. Participants must detect those that are the same as the one presented just before (e.g., the letter “J” in the sequence “A-K-J-J”). In the 2-back blocks, the letters are color-coded in red. Participants must detect letters that are the same as the one presented before the last (e.g., the letter “K” in the sequence “G-K-O-K”). Participants must indicate targets by pressing a button. Four 1-back and four 2-back blocks are presented interleaved. Between them, the baseline stimulus (an asterisk flashing with the same frequency as the letters) is shown for 16 s. Participants will undergo a training session outside of the scanner before starting.

To measure participants’ performance in the n-back, we will use the signal detection theory index of sensitivity, d′ (47), which separately analyses true and false positives. We will exclude participants with negative d′ values in either or both 1-back and 2-back versions of the task, as negative values suggest that participants are not performing the task effectively.

#### 2.5.2 Verbal memory paradigm

The verbal memory task (duration: 9 min 41 s) consists of an audio-adapted version of a well-established neuropsychological test, the Rey Auditory Verbal Learning Test (RAVLT) (48), which evaluates both encoding and retrieval verbal memory. In the control condition, patients passively listen to a list of incomprehensible words, then move onto the encoding phase where they must memorize a list of 15 understandable words. Following a silent period for retrieval, during which the patients must recall as many words as possible, the cycle is repeated. For quality assurance, participants will be asked to recall the words freely when finishing and outside the scanner. Instructions for that task will be given outside the scanner before starting and then just a reminder inside the scanner just before the task begins.

### 2.6 MRI data preprocessing

To estimate regional gray and white matter volumes from T1-weighted data, we will apply field bias correction, tissue segmentation, and normalization with SPM12 Segment and template creation and registration with DARTEL (49). We will use both modulated and unmodulated images as they convey complementary volumetric information (50) and apply 8 mm FWHM Gaussian smoothing. To estimate cortical thickness and surface area, we will use FreeSurfer (51) according to ENIGMA pipelines.^1^

To estimate WM tissue properties from DWI data, we will use the last generation BIDS App MRtrix3_connectome (52), which includes DWI denoising (53), Gibbs-ringing removal (54), geometric unwarping distortion corrections using TOPUP (55), eddy current and motion correction (56) and bias field correction (57). We will then use FSL “dtifit” to calculate the fractional anisotropy (FA), axial diffusivity, radial diffusivity, and mean diffusivity; we will also estimate microstructural diffusivity (58). For FA, we will establish a study-specific template by registering the FA images of all subjects to a common space and generating the mean FA skeleton, then project individual FA maps onto it and conduct Tract-Based Spatial Statistics (TBSS) (59, 60). Conversely, we will conduct voxel-based analyses for other diffusivity parameters.

Regarding spectroscopy, we will estimate absolute and creatine-corrected metabolite ratios in the volume of interest (VOI) located in the dorsomedial prefrontal cortex using LCModel version 6.3 (61). Previously, after visually confirming the correct positioning of the VOI, the T1-weighted sequences will be segmented with SPM12 (see above), and we will estimate within-voxel water concentration employing the retrieved tissue probability maps. This adjustment allows partial tissue volume correction when estimating the absolute metabolite concentrations. Further, we will fit glutamate, Glx (glutamate + glutamine), myoinositol, N-acetyl aspartate + N-acetyl-aspartyl-glutamate, and glycerophosphocholine + phosphocholine spectral peaks to a basis spectrum for absolute and creatine corrected metabolite quantification employing the unsuppressed water signal to perform eddy current correction. We will exclude metabolite levels showing FWHM (full-width at half-maximum) > = 10 ppm, SNR (signal-to-noise ratio) < 10, or SD (standard deviation of the estimated concentrations of each metabolite) > 15%.

Finally, we will preprocess fMRI data using fMRIPrep (62), which includes motion correction, slice time correction, susceptibility distortion correction using the GFM, 4 mm FWHM Gaussian smoothing, detrending, and high-pass temporal filter with a 128 s cut-off. Following preprocessing, we will conduct the first-level (individual) analysis to obtain activation maps by fitting a GLM to the preprocessed images using the Nilearn toolbox. We will design the model’s regressors to capture changes in the BOLD signal corresponding to each stimulus, convolving task-related temporal representations with the canonical Glover hemodynamic response function. Nuisance regressors derived from fMRIPrep will be incorporated into the design matrix to minimize the residual error. For n-back, we will compute the following contrasts: 1-back vs. baseline, 2-back vs. baseline, and 2-back vs. 1-back. We will compute the following contrasts for verbal memory: recall vs. control, encoding vs. control, and recall + encoding vs. control. Finally, utilizing the anatomical scan, the activation maps will be spatially normalized to a 2 × 2 × 2 mm^3^ MNI template by employing the ANTs registration toolbox in preparation for subsequent second-level analysis.

### 2.7 Data analysis

The primary outcome will be the correlation between pre-post changes in the activation of the dorsolateral and dorsomedial prefrontal cortex during the working memory n-back task (2-back vs. baseline) and pre-post changes in psychosocial functioning assessed with the Functioning Assessment Short Test (FAST) (63). Specifically, we will conduct three correlations: one for the left dorsolateral, one for the bilateral dorsomedial, and one for the right dorsolateral prefrontal cortex. We will use a permutation test to correct for multiple testing (three correlation tests).

For the secondary analyses, we will conduct two kinds of correlations: (a) pre-post changes in MRI data with pre-post changes in cognition and psychosocial functioning outcomes, and (b) baseline MRI data with pre-post changes in cognition and psychosocial functioning outcomes. We will include as covariates the variables from age, sex, bipolar disorder type, medication (use of lithium, antiepileptics, antidepressants, and antipsychotics), illness history (duration of the illness and number of episodes), or educational level that show statistically significant associations with the MRI signal or the cognition/functioning outcome; in case that several variables show statistically significant associations, we will select those still showing statistical significance in multiple regression. We will report results for structural MRI analyses using both familywise error rate (FWER) < 0.05 based on conservative permutation tests (64) and threshold-free cluster enhancement (TFCE) (65) and uncorrected *p* < 0.001 (at the exploratory level). For spectroscopy, we will use linear models. Finally, for fMRI analyses, a paired t-test will be conducted on single-subject contrast to examine temporal changes in activation, employing the Nilearn toolbox. We will break down and localize clusters using SDM ‘imgcalc’ tool (66).

We will also repeat all the analyses on those results that reach statistical significance according to sex to explore potential differences.

## 3 Discussion

Population aging and psychosocial functional impairment, commonly associated with BD, reinforce the need to develop interventions for this specific population, which are so far virtually non-existent, and identify potential neural correlates of this intervention to enhance our understanding of the underlying intervention-specific mechanisms. As far as we know this is the first study to investigate the functional brain correlates resulting from the functional remediation program specifically adapted to OABD (FROA-BD). It will also explore the potential role of MRI brain biomarkers as a potential predictor of cognitive or functional enhancement after receiving this specific intervention. Therefore, this study may contribute to identify neurocircuitry-based biomarkers that may be highly beneficial tools to optimize FROA-BD and promote more personalized treatments for each patient. We expect to find correlations between increased dorsolateral or dorsomedial activation in the n-back working memory task during FROA-BD and improved psychosocial functioning.

In recent years, several studies have investigated the brain effects of cognitive remediation using MRI techniques and whether neuroimaging can predict improved cognition or functioning in CR (28–31). However, the results are still scarce, and, to our knowledge, they are only focusing on younger people and none of them to date have investigated the brain effects in OABD.

Despite the FROA-BD neural substrates not being studied yet, our research team has already analyzed the neural markers that predict the efficacy of other psychotherapies. For instance, the frontal areas activation involved in salience and interoception processing, including the dorsomedial prefrontal cortex, predicted improvement with cognitive-behavioral psychotherapy in anxiety-related disorders (67). Miskowiak et al. (31) found that pre-treatment dorsolateral prefrontal hypoactivation predicted greater improvement in executive function after 10 weeks of action-based cognitive remediation in patients with BD. They also found an early dorsolateral prefrontal activity change during the working memory task that predicted improved executive functions after treatment completion (29). The same research group investigated not only associations between pre-treatment functional brain activation and response to treatment of cognitive remediation, but also explored the association between pre-treatment structural brain measures and treatment success. Specifically, they found that a minor baseline dorsolateral prefrontal cortical thickness was associated with greater treatment efficacy on executive functions, contrary to what they expected (30). In addition, vertex-based analysis also revealed an association between smaller superior temporal gyrus volume at pre-treatment and improved executive function after receiving cognitive remediation.

Concerning verbal learning and memory, the neural correlates of treatment-related improvements remain unclear, with a dearth of studies and conflicting findings (30, 68, 69). In this sense, some studies used a picture encoding task, an fMRI paradigm that probes visual memory for investigating the neural correlates of verbal memory improvement. Another study conducted in BD and major depressive disorder, after 10 weeks of cognitive remediation demonstrated an increase in the activation in lateral and medial prefrontal, superior temporal and lateral parietal regions in the n-back tasks and an activation increased in the bilateral hippocampus during the memory task (28). In our protocol, we will use a verbal memory encoding fMRI paradigm based on the RAVLT, with verbal material (auditory), which could be a highly sensitive verbal learning paradigm and more able to elicit hippocampal activation (70).

In addition, exploring the potential differences in treatment response or neural correlates based on sex could be valuable. For that reason, we will carry out exploratory analyses separately. We will repeat all the analyses on those results that reach statistical significance according to sex to explore potential differences depending on sex. To date, there is limited evidence suggesting a gender effect on memory-related functional activation (71–73) and the research examining sexual differences in the neural substrates of recall memory is inconclusive (74, 75). Regarding the efficacy of cognitive remediation in mood disorders no clear effect of sex has been found in treatment response (76). Some clinical manifestations are different between sexes in OABD, where females scored higher on anxiety and hypochondriasis, and had a higher number of hospitalizations. In contrast, males had more lifetime substance abuse disorders (77) suggesting the existence of different clinical and disease underlying mechanisms between the two sexes. Other potential confounding variables, in addition to age and sex, that will be considered in the analysis, could be pharmacological treatment, illness history, and educational attainment due to the association with cognitive performance, response to treatment, and brain functionality.

This project has potential limitations. First, we expect a loss of participants during the intervention or the follow-up period, mainly due to the higher frequency of different medical comorbidities usually presented by this group of patients. Although Nuclear Magnetic Resonance is a safe procedure that does not involve radiation emission, it may lead to a loss of subjects in the study due to contraindications such as patients with a pacemaker or any metal prosthesis, panic attacks and agoraphobia or claustrophobia. Nevertheless, we expect a reduced number of dropouts given that most of the patients will be followed in our unit. The possibility of participant attrition has also been considered in the sample size calculation. Besides, other approaches have been considered to address this risk, such as expanding the participants’ recruitment to other local mental health centers. Lastly, including not only patients in full, but also in partial remission, with a more representative real-world population in the daily clinical practice, will also contribute to limiting this potential issue (78). We acknowledge that a RCT design would also provide information about the differences in brain changes between the individuals following the FROA-BD program and those in a control group. However, in this study, we were not interested in the differences in brain changes between the FROA-BD and a control group but in the brain changes associated with a higher efficacy among individuals following the FROA-BD program. Another potential limitation of the study is the lack of resting state fMRI. However, we included MRI sequences based on previous works about cognitive remediation in BD.

This project aims, for the first time, to contribute knowledge on the neural underpinnings of functional remediation for OABD, to optimize this psychotherapy. Consequently, the project’s outcomes will establish the knowledge for enhancing functional remediation, potentially benefiting this group of patients. The findings are expected to directly improve psychosocial functioning and quality of life for this population. As emphasized by the International Society for Bipolar Disorders (ISBD) (5, 78), assessing the impact of novel treatments on the brain, such as through MRI, can provide knowledge in identifying specific changes in function and structure associated with cognitive performance and, in turn, may then offer insights into the potential effectiveness of the treatment. In fact, the use of neuroimaging could yield several additional benefits. On the one hand, it could provide information on how cognitive rehabilitation modulates different brain functions, offering insights into optimizing this psychotherapy. On the other hand, baseline MRI could serve to predict, for each individual, the extent of improvement in cognition and functioning in cognitive remediation. Identifying key targets associated with improved treatment response, on one side, and the neural correlates underlying cognitive function, on the other side, contributes to designing strategies aimed at precision and personalized medicine. This approach can help provide specific tools to patients, ensuring a tailored benefit based on their individual characteristics.

In conclusion, this study aims to investigate the neural mechanisms underlying FROA-BD, a specific intervention designed to improve functional and cognitive outcomes in older adults with BD. We will use different structural and functional magnetic resonance imaging to provide insight into its neural mechanisms. We consider the results of this study can provide a basis for developing personalized treatments in the elderly population and contribute to optimizing the design of the therapy itself.

## 4 Ethics and dissemination

This project has been approved by the Ethical Committee of the Hospital Clinic of Barcelona. We will follow the ethical principles of the Declaration of Helsinki and Good Clinical Practices. All participants will receive comprehensive details about the study, and will provide written informed consent before enrolling in the study. Participants who complete the study will receive financial compensation for the inconvenience and time spent on assessment visits.

The results obtained in this project will be disseminated among the scientific community through specialized scientific publications and conferences within the field of neurosciences and psychiatry to ensure the transmission of knowledge. The results of the project will also be shared among the general public through institutional websites and social media.

## Ethics statement

The study was approved by Ethics Committee of Hospital Clinic of Barcelona. The studies will be conducted in accordance with the local legislation and institutional requirements. The participants will provide their written informed consent to participate in this study.

## Author contributions

LM: Writing – original draft, Writing – review & editing, Conceptualization, Investigation. BS: Writing – original draft, Writing – review & editing, Conceptualization, Investigation. LF: Writing – review & editing. EJ: Writing – review & editing, Conceptualization, Investigation. AM-A: Writing – review & editing, Conceptualization, Investigation, Methodology. EM-H: Writing – review & editing, Conceptualization, Data curation, Investigation, Methodology. JS-M: Writing – review & editing, Conceptualization, Investigation. MO: Writing – review & editing, Methodology. JP: Writing – review & editing, Data curation, Methodology, Software. AS: Writing – review & editing, Methodology, Software. CT: Writing – review & editing, Conceptualization, Investigation. EnV: Writing – review & editing, Data curation, Investigation, Methodology. MP: Writing – review & editing, Methodology. EdV: Writing – review & editing, Conceptualization, Investigation. JR: Writing – review & editing, Conceptualization, Data curation, Funding acquisition, Investigation, Methodology, Project administration, Software, Supervision.

## Footnotes

^1^https://enigma.ini.usc.edu/protocols/imaging-protocols/
